# Evaluation of the Joining Response of Biodegradable Polylactic Acid (PLA) from Fused Deposition Modeling by Infrared Laser Irradiation

**DOI:** 10.3390/polym12112479

**Published:** 2020-10-26

**Authors:** J. M. Vazquez-Martinez, D. Piñero, J. Salguero, M. Batista

**Affiliations:** Department of Mechanical Engineering & Industrial Design, Faculty of Engineering, University of Cadiz, Av. Universidad de Cadiz 10, E11519 Puerto Real-Cadiz, Spain; david.pinero@uca.es (D.P.); jorge.salguero@uca.es (J.S.); moises.batista@uca.es (M.B.)

**Keywords:** additive manufacturing, polylactic acid (PLA), fused deposition modelling (FDM), laser joining, tensile strength

## Abstract

The development of high-complexity geometry parts is one of the main goals of additive manufacturing technology. However, the failure of printed structures and the joining of different parts to create complex assemblies represents a real challenge in the research of efficient and sustainability techniques for the permanent assembly of polymers. Laser welding processes have been used as a single-step method to join metals for years. Nowadays, the growing trend in the use of thermoplastics for additive manufacturing has led to the need to adapt this technique to materials with a very specific nature and which are more sensitive to thermal effects. In addition, the possibility of transmitting the laser beam through transparent polymer layers allows to us focus the energy supply on internal sections of the assembled components. In this research, an infrared laser marking system was used to join two different samples of polylactic acid manufactured by fused deposited modeling technology. In order to increase the effectiveness of the bonding process, a transparent and a dark sample have been used as assembly material, focusing the laser beam on the interface area of the two parts. By means of tensile tests, dimensional measurement and the use of optical microscopy techniques, a basis was established that links the supplied energy by laser to the joining performance.

## 1. Introduction

Plastics have become attractive materials in several industrial fields through the orientation to innovative development processes with a lower quantity of components. This fact allows for improving the assembly stages and reducing material incompatibilities. In the case of polymers for 3D printing applications, the use of new materials such as polylactic acid (PLA), with excellent thermoplastic and biodegradable properties, makes the process of great interest in a wide range of scientific areas, ranging from biomechanical solutions to high-impact industrial resources [1,2,3,4]. Furthermore, the possibility to improve the physicochemical and mechanical properties of PLA by the use of additives and reinforcements is one of the most investigated research lines to extend the application range of this material. Those considerations mean that polylactic acid, as a biodegradable material, plays an important role in replacing the petrochemical-based polymers commonly used in medical and industrial applications [5,6,7,8,9,10].

One of the main aspects of the growth in the global consumption of PLA for additive manufacturing processes is based on the mechanical properties shown by this material, that can range from soft and elastic elements to high-strength parts. This ability to adapt the properties can be observed in the tensile modulus range (0.35–3.5 GPa). In addition, the relatively low melting temperature (150–162 °C) results in a highly versatile material for the development of functional parts [5].

The growing trend in the use of fused deposition modelling (FDM) systems in a wide range of applications is highly related to the improvement in the accuracy of the printed parts. However, higher volume printers show lower accuracy in dimensions and geometrical tolerances than medium- and small-range volume printers. For this reason, most of the industrial applications based on FDM systems use medium-size printers (<1 m^3^) for the development of plastic parts [11]. 

Although a significant variety of geometries and sizes can be manufactured using PLA as the base material, most components cannot be manufactured as a single part due to the complexity or the size of the elements. For this reason, joining of sub-components is often required by permanent or removable joining procedures. Until now, one of the most widely adopted technologies for this objective has been based on the use of chemical adhesives. However, these adhesive processes often show some characteristics that can reduce the environmental sustainability of the process [6,12,13]. Furthermore, the use of some adhesives with corrosive nature may reduce the life of PLA components, also preventing the recycling of used parts. As an alternative to the use of chemical adhesives, some authors suggested an innovative research line based on the joining of PLA components by the supply of focused energy over reduced areas. For this purpose, the use of laser techniques can achieve the heat transfer control for the melting of localized areas of plastic materials, facilitating the bonding process of both elements [14,15,16,17,18].

Laser irradiation processes have been widely used as surface modification treatments to improve the roughness of additive manufacturing (AM) parts. Through the melting of the highest asperities, the irregularities in the printing process can be reduced, resulting in a more uniform surface [19,20,21]. The characteristics of the laser system makes it possible to concentrate high energy ranges on very small areas, increasing the accuracy of this method. In addition, the great variety of control parameters that can be used for modifying the energy supply properties to the target surface allows us to adapt the laser treatment to high specific work conditions. For this reason, laser joining technology (LJT), including laser welding techniques, is particularly efficient in those applications where the chemical components of conventional adhesives cannot be used (corrosive environments, biomedical components, food applications). 

The nature of LJT, based on light amplification, implies variations in the beam transmission for different reflective materials. Under this consideration, the ability to supply a large amount of energy through transparent layers places the laser treatments in advance of other bonding technologies that needs to separate the surfaces to be joined. Although some alternatives can be performed to carry out this process, the layers of the elements subjected to the joining process usually needs to be positioned in a specific way to ensure that the laser beam can pass through the upper layers. This fact implies that transparent PLA should be placed before other color layers that favors the absorption of the irradiation energy [18,22,23,24]. In addition, the application of thermal processes by laser avoids the need to prepare/activate the surfaces by means of chemical or mechanical procedures. 

The aim of the present work was to investigate the effect of laser processing parameters on the joining properties when the PLA additive manufacturing polymer is irradiated. Nine different sets of black and colorless PLA samples with variations in the energy density of pulse were tested and characterized. The joining performance was related to their maximum tensile strength after the laser irradiation process; moreover, the absence of deformation over the treated surfaces was considered as an objective to validate the quality of this procedure. Potential applications to repair damaged manufactured parts have been explored through the calculation of equivalent section dimensions for the replacement of full fill density parts. 

## 2. Materials and Methods 

### 2.1. FDM Printing Process

The experimental methodology to evaluate the joining properties of the laser irradiation process considers three different probes with sizes and geometries adapted to specific evaluation conditions. 

Joining probes were manufactured by polylactic acid (PLA) filaments with 1.75 ± 0.03 mm diameter from FFFWorld (Cantabria, Spain). A handmade modified fused Deposition Modelling 3D printer Cube X Duo from 3D Systems (Santa Clarita, CA, USA) was used. Printing parameters shown in Table 1 were selected to develop upper and lower parts, under 20 ± 1 °C and 60 ± 10% relative humidity environmental conditions. 

**Figure 1:**
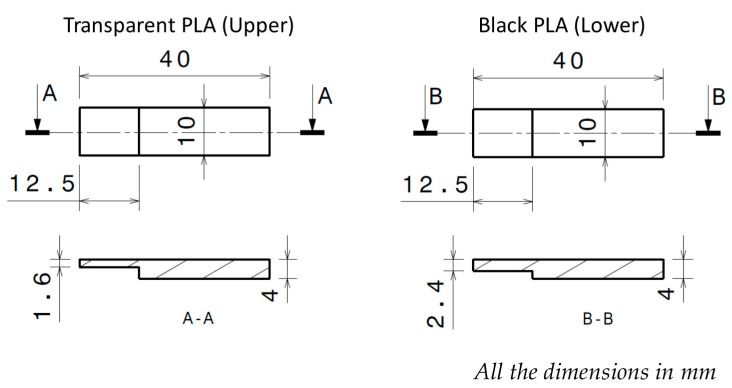
Polylactic acid (PLA) probe design for laser irradiation joining.

Two different parts were designed with the dimensions shown in Figure 1 to optimize the welding ability of the process, maintaining an overlapping area between upper and lower probe. In addition, to facilitate the light transmission through the upper part, focusing the beam energy on the contact area of the joining probes, transparent PLA tonality was used for the manufacturing of the upper part and black PLA for the lower part.

With the main purpose of obtaining a section of a 3D-printed PLA part that corresponds to the axial efforts of the laser joining, a new type of probe was designed. Cylindrical samples with variation in the cross-sectional area were manufactured with the same printing parameters as joining probes previously described, maintaining 100% of the value of fill density.

### 2.2. Laser Irradiation for PLA Joining Process

**Figure 2:**
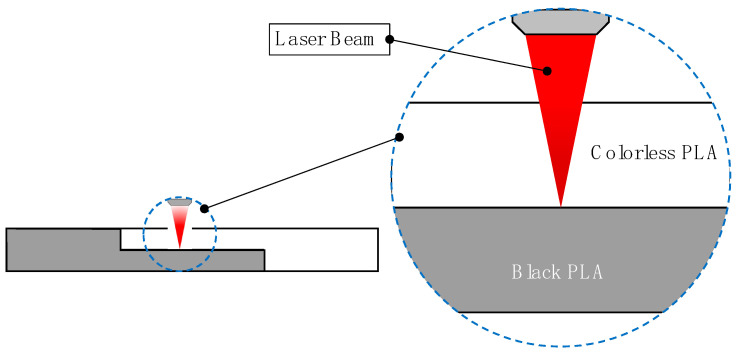
Laser beam focus on the interface of the colorless and black joining probes.

The laser joining process of the printed parts was carried out by the irradiation of a 5 × 5 mm area focused on the overlapping interface between the colorless and black probes, as shown in Figure 2. All irradiation processes were performed using a 20 W ytterbium-fiber infrared laser system (ROFIN-SINAR Technologies Inc., Plymouth, MI, USA). Spot diameter and pulse width were 60 µm and 100 ns, respectively. 

The irradiation of the joining areas was performed at nine different pulse energy densities (Ed), using the same pulse rate and scanning speed for all the samples. Energy density was altered by selecting different laser power values (Table 2). Energy density decreases as laser power decreases, giving place to a reduction in the aggressiveness of the joining process. All the irradiation treatments were carried out through linear bidirectional layout with a 0.1 mm separation between laser tracks under an open-air atmosphere.

On the one hand, the effects of the laser irradiation process on the PLA surfaces were evaluated by optical microscopy techniques to identify specific defects of heat transfer incidence. In addition, geometrical and dimensional variations were measured using an electronic comparator set, with a 0.0001 mm resolution. Measurement of the thickness and cross-section were carried out to evaluate the incidence of the joining treatment on the resulting size of the studied parts. On the other hand, the joining strength to the failure of the laser irradiation were evaluated by standard tensile test, adapted to the PLA polymer features and the dimensions of the probes. The tensile test was performed following the UNE-EN 1465:2009 (ISO 4587:1979) standard using a Shimadzu Autograph AG-X tensile testing machine (Shimadzu Corporation, Kioto, Japan). Specific flat grips (SCG-5KNA) for soft materials as plastics were used, with 2 mm/min test speed.

### 2.3. Corresponding Size Section that Can Be Replaced by Joined Parts 

**Figure 3:**
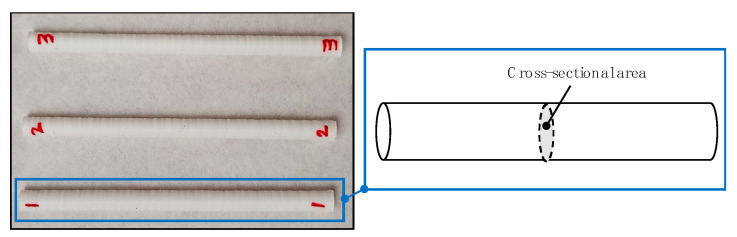
Cylindrical printed parts for corresponding section that can be replaced by joining.

In order to estimate the range of the parts that can be replaced by joined sets, a new type of test probe was designed based on the size of 100% density fill of PLA cylindrical parts. These probes were developed to calculate the equivalent cross-sectional area of a cylindrical part that can ensure the maximum tensile strength of laser irradiation joining. Taking as reference the printing parameters of the joining samples, the variation in the cross-sectional area of the cylindrical elements was taken as the control parameter to evaluate the maximum tensile strength behavior as a function of the size of the printed samples (Figure 3). Manufacturing parameters of equivalent section cylindrical parts are shown in Table 3. 

## 3. Results

Although all the joined parts failed in the overlapping area, the experimental results show a significant influence of the laser radiation parameters on the properties and surface integrity of the joined samples. The use of laser techniques implies variations in the maximum tensile strength, also affecting the maximum deformation range of the probes under axial efforts. As a consequence of the high energy supplied on a reduced focus area, some defects may be caused on the surface of the joining samples, decreasing the functional performance of the process.

### 3.1. Effects of Laser Irradiation on Tensile Properties of Joining Parts

**Figure 4:**
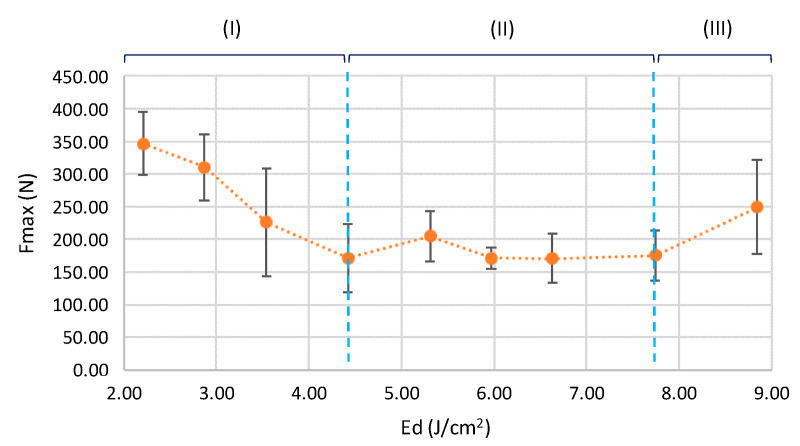
Maximum tensile strength of joined PLA as a function of energy density of pulse.

The energy density of pulse (Ed) was considered as control parameter for the application of laser radiation treatments in the joining process. Increasing the Ed on the joining area results in the development of three different behavior ranges of joined parts as a function of the tensile strength. Each range is mainly characterized by a behavioral trend that provides specific characteristics to the joining process. As shown in Figure 4, these trends were easily detected by a tensile test of the joined samples, where the values of maximum strength (Fmax) and maximum deformation (Dmax) were affected.

The first range (I) developed under lower energy density values implies a significant decrease in the maximum tensile strength when increasing the Ed. The second set (II) observed is characterized by the uniformity of Fmax values, maintaining a trend without significant variations in the behavior of tensile strengths. However, a slight growth can be observed in the last stage of the graph (III) for higher Ed evaluated. These variations on the maximum tensile strength behavior are highly related to the cooling process on different melted volumes of PLA under radiation treatments. The increase in supplied energy from the laser beam causes a growth of the thermally affected zone (TAZ) of the contact surface of joined parts, especially in the lower sample (Black PLA), increasing the molten material volume. The higher amount of molten material generates instabilities in the cooling stage, giving rise to a lack of uniformity in the re-solidification process between contact surfaces. In Stages II and III, a significant increase in the molten material volume was detected that results in the collapse of the colorless sample (upper). This effect means that the melted material flows out of the assembly set, also making it difficult to keep the irradiated PLA over the joining area, preventing the filling of the micro-holes produced by the laser pulses, and thus reducing the integration of the materials of both parts (Stage II). The last stage of Figure 4 (Stage III) shows a slight growth in the Fmax value, mainly due to the increase in the dimensions of the crater of the lower part (dark PLA). This phenomenon means that the melted material of the collapsed upper part (colorless PLA) penetrates into the crater, increasing the cohesive force between samples.

### 3.2. Effects of Laser Irradiation on Surface Integrity of Joining Parts

**Figure 5:**
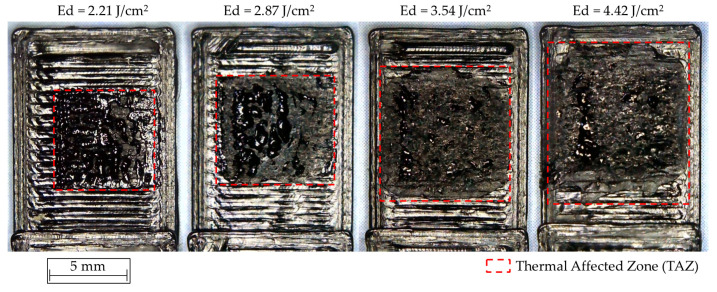
Thermally affected zone in the lower probe (Black PLA) as a function of Ed.

As described previously, more aggressive treatments with higher Ed values are associated with a greater incidence of the laser beam on the contact surface between the parts to be joined. As a consequence of the radiation energy supplied, a significant increase in the area of molten material considered as TAZ is caused in the lower sample, as shown in Figure 5.

**Figure 6:**
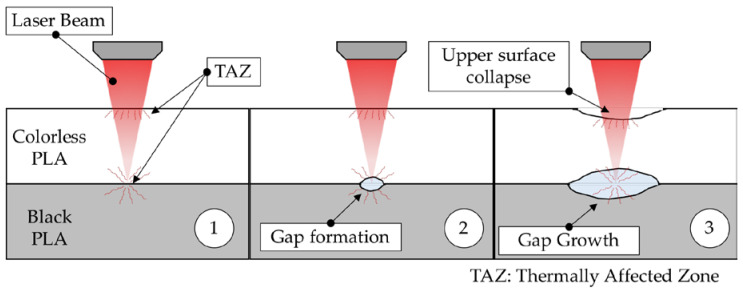
Thermal effects on the interface of the PLA samples for low, medium and high Ed.

This behavior is also associated with an increase in the depth of incidence of the beam on the lower sample, which causes the collapse of the surface and the formation of a crater. The location of the focus point of the laser beam between lower and upper joining probes also induces radiation heat transfer to each surface, resulting in deformation towards the internal layers of the black and colorless samples, as shown in stage 1 of Figure 6. The increase in the supplied energy (Ed) leads to the growth of the generated gap, reducing the joined section to the outside edge of the irradiated areas, as shown in stages 2 and 3 of the Figure 6. These increases in the size and depth of craters cause a decrease in the effectiveness of the bonding zone between PLA surfaces, thus decreasing the tensile Fmax. 

For more aggressive treatments, the combined effect of heat transfer with the incidence of the beam through the colorless sample can also affect the furthest section of the probe, causing the collapse of the surface, as shown in stage 3 of Figure 6. This type of defect may be considered as external defect in the joined parts, evaluated as a lack of the quality of the manufactured parts.

The joining process of elements manufactured in PLA has as its main objectives the assembly of complex geometries, and the repair of printed components. For this reason, the laser joining process must be carried out on reduced sections of components, and maintaining an important control of the processing parameters. This consideration leads to preventing the appearance of defects and ensure the structural integrity of the elements involved. The maximum deformation of upper surface (colorless PLA) by the radiation effects (Ds) was directly measured by an electronic comparator set.

**Figure 7:**
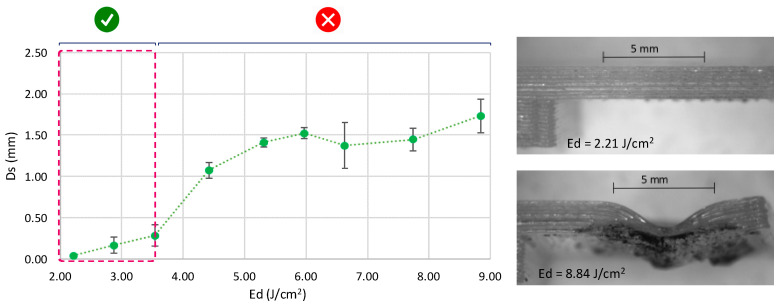
Upper surface deformation of irradiated samples for joining: not thermally affecting external surfaces (√) and external layers of the sample (x).

Upper surface collapse by the irradiation effect causes a deformation that can be considered as a quality requirement to validate the use of the technology. Under this consideration, the supplied energy, in terms of the energy density of the pulse of the laser treatment, shows a significant influence in the deformation of the upper surface of the joined parts. In this case, it has also been observed that increases in energy density per pulse result in greater deformation of the irradiated section, showing a critical growth from 3.54 J/cm^2^, as shown in Figure 7.

Under this behavior, two different groups of treatments can be considered regarding the deformation of the sample surface. On the one hand, lower energy density involves lower deformation and may be considered as not thermally affecting external surfaces (√). On the other hand, higher energy treatments imply an excess of the molten PLA, and as consequence, the collapse of the surface, resulting in large deformation of the external layers of the sample (x). As a result, an optimal range of pulse density energy lower than 4.00 J/cm^2^ for the use of laser joining technology may be confirmed. 

**Figure 8:**
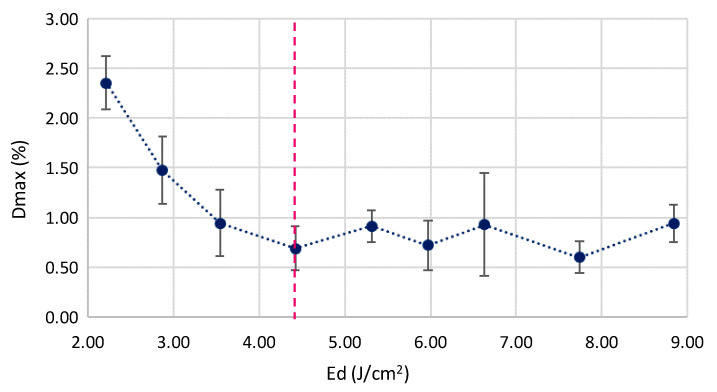
Maximum tensile deformation of joined parts as a function of Ed.

In addition to deformation phenomena on the thermally affected surfaces, the laser irradiation process may affect the printing structure of the PLA, with varying mechanical properties from the initial parts. The melting and cooling process of the overlapping samples interface modify the internal printing structure of each parts, mainly based on the variation in fill density on located sections (irradiated area). One of the main consequences of the variations in the internal structure of small sections is a reduction in the tensile maximum deformation (Dmax), measured by a tensile testing machine. This effect is magnified when increasing the melted material volume. Based on this consideration, a significant reduction (>220%) in the maximum tensile deformation until the failure of the joined part has been detected, as a function of the supplied energy (Ed). This reduction is maintained in a uniform range for Ed values over approximately 4.42 J/cm^2^, where critical volume of the melted material causes a similar behavior in the joined samples, as shown in Figure 8. 

**Figure 9:**
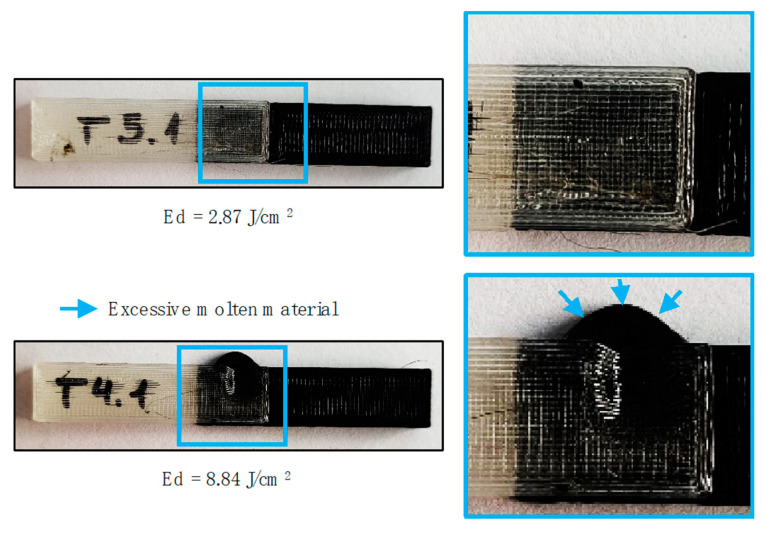
Excessive molten material for high density energy treatments.

An excess of melted PLA in the interlayer of the joined parts, combined with the developed gap caused by the increased process energy, causes the melting material to flow to the outside of the overlapping samples. This fact is especially evident in high energy density treatments where a significant volume of material (especially black PLA) solidifies between the joined parts, as shown in Figure 9.

### 3.3. Corresponding Features of Joining Replacing Parts

One of the main purposes of using laser joining processes is based in the replacement and repair of damaged PLA parts. For this reason, the understanding of the equivalent sections of printed elements that can be replaced by joined parts is one of the main objectives in this research. Taking as a reference the tensile strength of the 5 × 5 mm irradiated treatment, a study was carried out to calculate the section of a 100% density fill printed part that shows similar resistance to joined elements.

**Figure 10:**
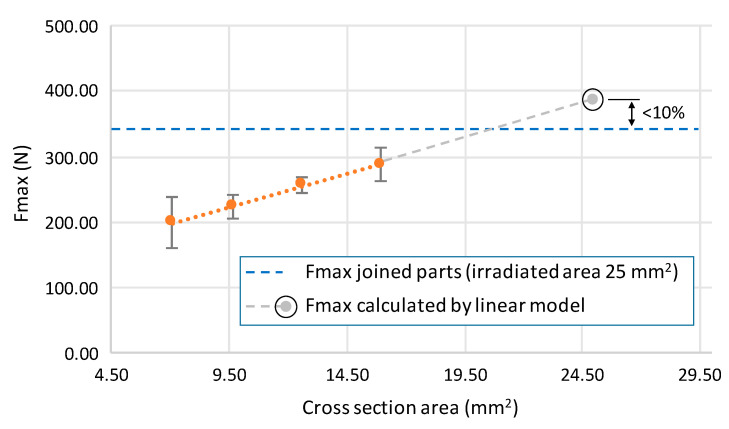
Fmax as a function of section area of equivalent printed parts.

The evaluation of maximum tensile strength on different sections of printed parts demonstrated that Fmax is highly influenced by the area of the sample, as confirmed by experimental tests. In Figure 10, a linear trend is detected, showing a proportional growth between both study parameters.

The increasing behavior of Fmax as a function of part section (A) is governed by the linear model of Equation (1), showing a determination coefficient (R^2^) higher than 99.9% that ensures the goodness of the adjustment. Based on the linearity of the tensile strength behavior as a function of the area of printed sections, the Fmax of equivalent irradiated area (5 × 5 mm = 25 mm^2^) was calculated to validate the experimental assumptions. As a result, a variation lower than 10% was confirmed between the calculated Fmax of the equivalent sections, of full density fill printed parts, and the Fmax value of the joined parts that fits with the joining conditions of Ed = 2.21 J/cm^2^.

**Fmax [N] = 10.124 × A [mm^2^] + 127.160** (1)

The proposed model, and its validation by experimental procedures, allows us to detect the optimum values of the laser irradiation parameters to ensure the highest resistance and minimum deformation of the joined parts. Under these processing conditions, all the tested samples show similar tensile strength to continuous full density fill printed parts, obtaining a first approximation to the calculation of replacement sections for damaged parts by laser joining. 

## 4. Conclusions

The effects of the laser processing parameter on the thermal joining behavior of polylactic acid from fused deposition modelling technology have been studied. Three different behavioral ranges of joined parts have been confirmed, in terms of maximum tensile strength (Fmax), as a function of the supplied energy density of pulse (Ed), reaching a maximum value of 346.54 N for 2.21 J/cm^2^ of Ed. Although a stabilization of the values of Fmax has been detected from 4.42 J/cm^2^ energy density, a significant increase in the thermally affected area as a consequence of the incidence of the beam for higher Ed values. An increase in the thermally affected zone produces an internal gap formation between assembled parts that may cause the collapse of the upper surface, mainly due to the excess of melting material. In addition, cooling processes of higher volumes of melting materials may reduce the tensile maximum deformation by up to 220%. Under this consideration, optimal conditions for joining process by infrared laser on transparent and black PLA parts have been obtained for the lower energy density tested, where higher Fmax and lower deformation of external surfaces were observed. Based on the linear behavior of the cross-sectional area of PLA manufactured parts under tensile efforts, in terms of Fmax, it may be confirmed that laser-joined sections of 25 mm^2^ showed similar tensile resistance (<10%) to 25 mm^2^ cross-sectional parts (100% fill density). 

## Figures and Tables

**Table 1:** Manufacturing parameters of printed parts.

Parameter		Parameter	
**Layer thickness (mm)**	0.20	**Filling density (%)**	100
**Extrusion width (mm)**	0.40	**Filling pattern**	Linear
**Extrusion temperature (K)**	483.15	**Feed rate (mm/s)**	30
**Board temperature (K)**	333.15	**Acceleration (mm/s^2^)**	1000

**Table 2:** Laser irradiation parameters for joining process.

Laser Power (W)	Pulse Rate (Hz)	Scanning Speed (Vs)	Energy Density of Pulse (J/cm^2^)	Irradiated Area (mm^2^)
5.0	80,000	50	2.21	25
6.5			2.87	
8.0			3.54	
10.0			4.42	
12.0			5.31	
13.5			5.97	
15.0			6.63	
17.5			7.74	
20.0			8.84	

**Table 3:** Manufacturing parameters of printed parts for the study of equivalent section.

Parameter		Parameter	
**Layer thickness (mm)**	0.20	**Filling density (%)**	100
**Extrusion width (mm)**	0.40	**Filling pattern**	Linear
**Extrusion temperature (K)**	483.15	**Feed rate (mm/s)**	30
**Board temperature (K)**	333.15	**Acceleration (mm/s^2^)**	1000
**Geometry of sample**	Cylindrical	**Cross-sectional area (mm^2^)**	7.07-9.62-12.57-15.90
